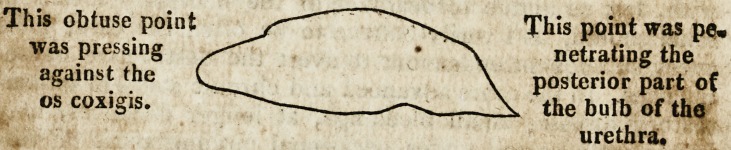# Case of a Bony Substance Extracted from the Rectum of a Young Man

**Published:** 1816-02

**Authors:** Lewis Henry

**Affiliations:** Madeira, Member of the Royal College of Surgeons, London.


### *For the London Medical and Physical Journal.*

*Case of a Bony Substance extracted from the Rectum of a Young Man;* by LEWIS HENRY, Esq. Madeira, Member of the Royal College of Surgeons, London.

WHEN the patient applied to me, I found him suffering under great constitutional irritation; his pulse 130, his countenance pale, cold sweats, frequent faintings, vomiting of a bilious matter, and frequent inclination to go to stool, with tenesmus. On examination of the rectum, the bone was found so strongly attached to the part, that with difficulty I extracted it with my dressing forceps; some bleeding succeeded, but of no moment; and, when the bone was extracted, my patient found himself apparently well. The next day, introducing a bougie up the urethra, I found that the point of it passed through an opening made by the bone. The peristaltic motion of the rectum could not have forced the bone to such a degree as to produce this violence: the bone must have penetrated the urethra by the strong action of the sphincter ani. The patient does not recollect ever swallowing the bone. Constitutional irritation must have been occasioned by the obtuse point of the bone pressing against the end of the great sympathetic nerve situated on the point of the os coxigis.

The bone is in my possession. It is an inch and a half in height, and a quarter of an inch in breath; it has a sharp point and acute edge, and the other extremity is also loose and irregular; the other edge is rough and spongy, with a small membranous substance attached to its acute point. I shall give here an outline of it.

This obtuse point was pressing against the os coxigis.

This point was penetrating the posterior part of the bulb of the urethra.

---

We are glad to find our correspondence renewed with Madeira since the departure of Dr. Andrews, and shall be thankful to Mr. Henry for further communications. Has he any thing to add, on the subject of the leprosy of that island, to what we have learned from Dr. Adams?—EDIT.

## Figures and Tables

**Figure f1:**